# Acute kidney injury in the context of staged revision arthroplasty and the use of antibiotic-laden cement spacers: a systematic review

**DOI:** 10.1186/s13018-023-03809-2

**Published:** 2023-05-09

**Authors:** Yash P. Chaudhry, Mark LaGreca, Hunter Hayes, Efstratios Papadelis, Sandesh S. Rao, Raj Amin

**Affiliations:** 1 Department of Orthopaedic Surgery, Philadelphia College of Osteopathic Medicine, Philadelphia, PA USA; 2 Department of Orthopaedic Surgery, Washington Orthopaedics and Sports Medicine, Washington, DC USA; 3 Department of Orthopaedic Surgery, University of California San Francisco Fresno, Fresno, CA USA

**Keywords:** Acute kidney injury, Prosthetic joint infection, Antibiotic cement spacer

## Abstract

**Background:**

The most common cause of revision arthroplasty is prosthetic joint infection (PJI). Chronic PJI is commonly treated with two-stage exchange arthroplasty involving the placement of antibiotic-laden cement spacers (ACS) in the first stage, often containing nephrotoxic antibiotics. These patients often have significant comorbidity burdens and have higher rates of acute kidney injury (AKI). This systematic review aims to assess the current literature to identify (1) AKI incidence, (2) associated risk factors, and (3) antibiotic concentration thresholds in ACS that increase AKI risk following first-stage revision arthroplasty.

**Methods:**

An electronic search was performed of the PubMed database of all studies involving patients undergoing ACS placement for chronic PJI. Studies assessing AKI rates and risk factors were screened by two authors independently. Data synthesis was performed when possible. Significant heterogeneity prevented meta-analysis.

**Results:**

Eight observational studies consisting of 540 knee PJIs and 943 hip PJIs met inclusion criteria. There were 309 (21%) cases involving AKI. The most commonly reported risk factors included perfusion-related factors (lower preoperative hemoglobin, transfusion requirement, or hypovolemia), older age, increased comorbidity burden, and nonsteroidal anti-inflammatory drug consumption. Only two studies found increased risk with greater ACS antibiotic concentration (> 4 g vancomycin and > 4.8 g tobramycin per spacer in one study, > 3.6 g of vancomycin per batch or > 3.6 g of aminoglycosides per batch in the other); however, these were reported from univariate analyses not accounting for other potential risk factors.

**Discussion:**

Patients undergoing ACS placement for chronic PJI are at an increased risk for AKI. Understanding the risk factors may lead to better multidisciplinary care and safer outcomes for chronic PJI patients.

Level of evidence III.

## Introduction

As total joint arthroplasty (TJA) becomes more prevalent in the USA, the burden of revision arthroplasty is expected to increase as well [1]. One of the most common reasons for revision arthroplasty is prosthetic joint infection (PJI) [1, 2], which is associated with significant morbidity and mortality [3, 4] An estimated ~ 1% of all total joint arthroplasties (TJA) result in a PJI, but it has been suggested as the driving factor behind the recent increase in revision burden [5]. Two-stage revision arthroplasty is considered by many the gold standard for treatment of a chronic PJI, particularly in the USA [6]. The first stage of a revision arthroplasty includes placement of an antibiotic-impregnated cement spacer (ACS), often containing nephrotoxic antibiotics such as vancomycin and tobramycin [7]. Two-stage revision arthroplasty has been shown to be highly effective in eradicating PJI in over 80% of cases [8]. Despite this success rate, it is associated with substantial morbidity and an alarmingly high mortality rate [3, 9]. This population tends to have a higher comorbidity burden which predisposes them to longer hospital stays, increase in overall health care dollars, and greater complication rates.

One such complication is acute kidney injury (AKI), which has been extensively studied in primary TJA but less so in the two-stage revision arthroplasty setting [10, 11]. This is clinically important because AKI carries a significant increase in patient morbidity and mortality [12]. It is important for both orthopedic surgeons and medical clinicians to be made aware of these risk factors and better help optimize patients and lead to safer outcomes for patients with chronic PJIs, particularly in a vulnerable population undergoing implantation of ACS that contain nephrotoxic agents.

The goals of this systematic review were to assess the existing literature to (1) establish an incidence of AKI following first-stage exchange arthroplasty, (2) identify perioperative risk factors for AKI risk, (3) determine the antibiotic concentration thresholds in ACS over which AKI risk is significantly increased.

## Methods

### Literature search strategy

This systematic review was conducted in accordance with the PRISMA (Preferred Reporting Items for Systematic Reviews and Meta-Analyses) guidelines [13]. An electronic search was conducted of the PubMed database of all studies published from inception to 1/21/2022 (see search terms in Appendix). Medical Subject Headings and Emtree terms were utilized to improve search results.

### Eligibility criteria

Studies were included for review if they (1) involved subjects undergoing ACS placement in the first stage of treatment for PJI, (2) included AKI as an outcome, as defined by either the Kidney Disease Improving Global Outcomes (KDIGO) [14] or the Risk, Injury, Failure, Loss of kidney function, and End-stage kidney disease (RIFLE) [15] classifications, and (3) had *N* ≥ 10 (case series and case reports excluded).

### Study selection

All screening and text review was conducted independently by two authors (YPC and ML). All disagreements were resolved through discussion among screening authors, with senior author involvement included as needed (RMA).

### Data extraction and synthesis

All data extraction was performed by three authors (YPC, ML, and HH). Extracted variables included country in which the study was conducted, study inclusion and exclusion criteria, patient characteristics (age, BMI, sex, preexisting chronic kidney disease [CKD], baseline serum creatinine level), procedure type (knee vs. hip revision), cement spacer type, antibiotic impregnated in the spacer, quantity of antibiotic used, systemic antibiotics administered during the study period, AKI definition, and incidence of AKI. Risk factors for AKI based on univariate and multivariate analysis were also extracted, as well as the subsequent odds ratios (OR) with 95% confidence intervals (CI). Given the high heterogeneity in antibiotics and spacer types, a meta-analysis could not be performed.

### Quality appraisal

Quality appraisal was performed using the Methodological Index for Non-Randomized Studies (MINORS) criteria for non-randomized studies, which is scored out of a maximum 16 points for non-comparative studies and 24 points for comparative studies [16]. This was performed independently by two authors (YPC, HH).

## Results

### Study characteristics

**Fig. 1:**
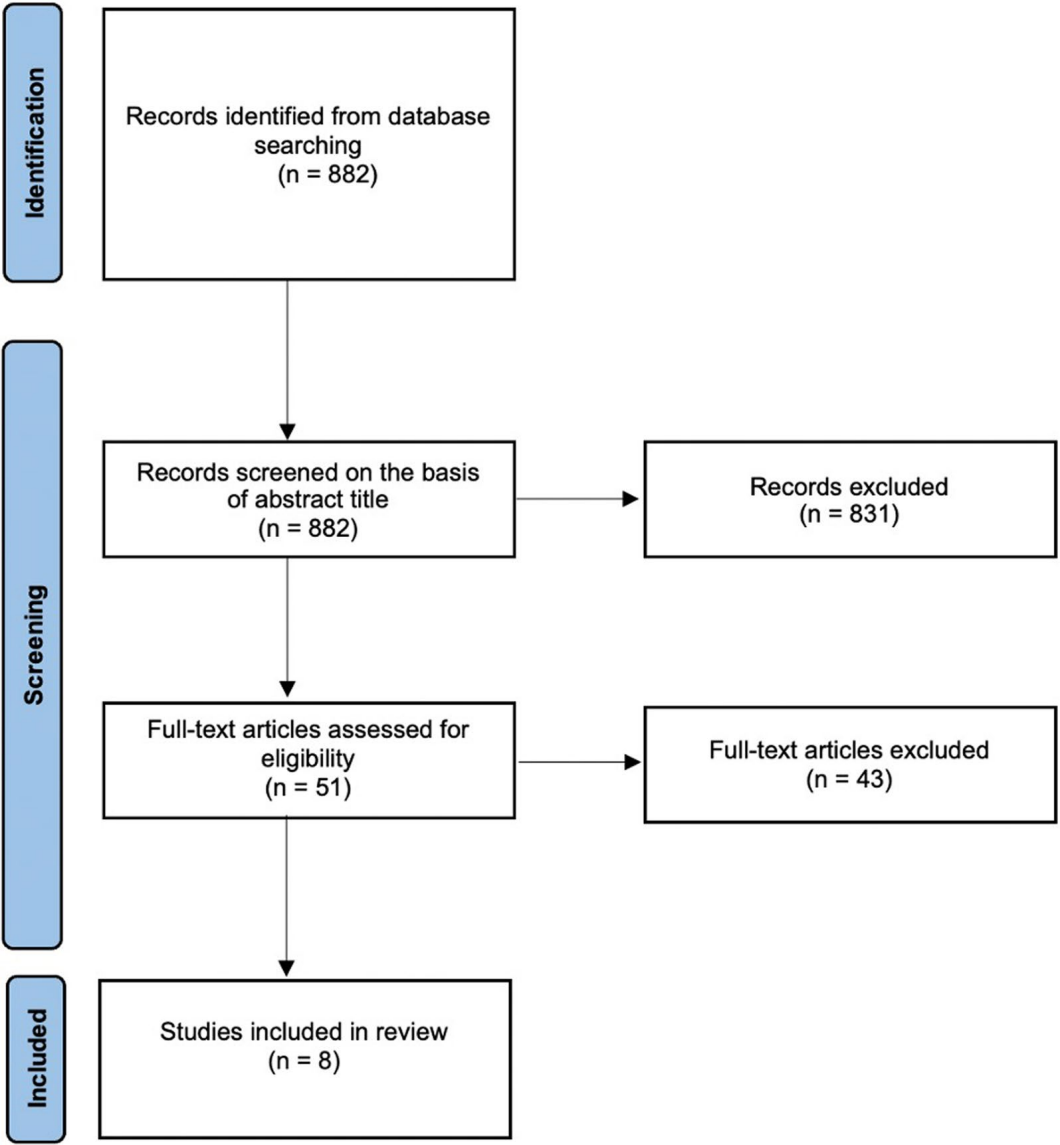
Preferred Reporting Items for Systematic Reviews and Meta-Analyses (PRISMA) flowchart

A total of 882 citations were identified (Fig. 1) and screened for inclusion. Fifty-one articles were identified for full text review, with 8 meeting the criteria for inclusion [17–24] (Table 1). Six studies were conducted in the USA [18–23]. The remaining two were conducted in Canada [17] and Germany [24]. This included 540 knee revisions and 943 hip revisions. Three studies included only knee revisions [18, 20, 23], one included only hip revisions [19], and the remaining four had both [17, 21, 22, 24]. ACS types, quantity of antibiotics added, and systemic antibiotic use are reported in Table 2. The incidence of AKI within this population was 21% (309 cases) (Table 3). The range of AKI incidence in the included studies was 10–33%. Two studies split up cases on the basis of preexisting CKD, finding higher rates of AKI in those with preexisting CKD (45% vs. 14% in 455 knee revisions [20], 28% vs. 7% in 256 hip revisions [19]).

**Table 1:** Descriptive characteristics of eight studies assessing rates of acute kidney injury in first-stage revision arthroplasty involving the use of antibiotic cement spacers

Study	LoE	Country	Age (years) mean (SD)	BMI (kg/m^2^) mean (SD)	Female (%)	CKD (%)	Pre-op Cr mean (SD)	*N* (%)		Inclusion criteria	Exclusion criteria
								Hip ACS	Knee ACS		
Aeng [17]	3	Canada	66 (13)	NR	NR	NR	0.86 (0.79–1.01)*	32 (64)	18 (36)	Age > 18, ACS with tobramycin powder + / − vancomycin powder in PMMA cement for first-stage revision of infected TKA/THA	Hemodialysis dependence or development of AKI prior to surgery
Berliner [18]	3	USA	67 (11)	31.3 (7.3)	48	NR	0.95 (0.53)	0 (0)	75 (100)	Static or articulating ACS for infected TKA	NR
Dagneaux [19]	3	USA	67 (11)	33 (8.0)	47	72 (16)	1.0 (0.5)	0 (0)	455 (100)	Chronic PJI following TKA treated with 2-stage exchange involving resection and ACS implantation	Total femoral constructs, bilateral TKA PJI, simultaneous PJI of TKA and another joint, ACS implanted at a different institution, resection only
Dagneaux [20]	3	USA	65 (12)	30 (7.0)	45	41 (16)	1.0 (0.3)	256 (100)	0 (0)	Chronic PJI following THA treated with 2-stage exchange involving resection arthroplasty and ACS implantation	Total femoral constructs, hemiarthroplasties, age < 18, bilateral hip PJI, simultaneous PJI of THA and another joint, ACS placed at different institution, resection only
Edelstein [21]	2	USA	67 (13)	32 (9.0)	41	NR	0.9 (0.2)	17 (46)	20 (54)	Infected primary TKA/THA treated with two-stage exchange involving resection arthroplasty and placement of ACS	Vancomycin/aminoglycoside allergy, no baseline Cr data, repeat spacer exchange required prior to second stage of revision, parenteral aminoglycoside given to reduce AKI risk unrelated to ACS
Geller [22]	3	USA	64 (12)	30 (7.0)	52	NR	1.0 (0.7)	91 (37)	156 (63)	Hip or knee PJI treated with two-stage revision procedure	Missing information on antibiotic type/dosage in ACS, missing preoperative Cr or postoperative hemoglobin
Menge [23]	3	USA	63* (NR)	NR	55	NR	0.9 (0.8–1.2)*	0	84 (100)	TKA resection and ACS placement, Cr value < 30 days before ACS placement, Cr value available within 90 days after ACS placement	NR
Theil [24]	3	Germany	NR	29* (NR)	52	NR	0.9 (0.7–1.2)*	144 (52)	135 (48)	Chronic PJI following TKA/THA treated with two-stage revision using PMMA ACS. PJI defined based on MSIS 2011 criteria	Single stage revision for early PJI or aseptic revision, reconstruction following tumor resection, no ACS placement in revision, Girdlestone procedures

*LoE* level of evidence, *SD* standard deviation, *BMI* body mass index, *CKD* chronic kidney disease, *ACS* antibiotic-laden cement spacer, *NR* not reported, *PMMA* polymethyl methacrylate, *TKA* total knee arthroplasty, *THA* total hip arthroplasty, *AKI* acute kidney injury, *USA* United States of America, *MSIS* Musculoskeletal Infection Society, *PJI* prosthetic joint infection, *Cr* serum creatinine

*Mean not reported; median reported in its place (interquartile range in parentheses)

**Table 2:** Descriptive characteristics of antibiotic cement spacers and systemic antibiotics in eight studies assessing rates of acute kidney injury in first-stage revision arthroplasty

Study	Spacer type	Antibiotic in spacer	Antibiotic quantity added	Postoperative systemic antibiotic
Aeng [17]	Plain Palacos (*n* = 34) or premanufactured with gentamicin (*n* = 16)	Premanufactured with 0.5 g/40 g cement	Tobramycin 3.6 g/40 g, 92% also received vancomycin 1.5 g/40 g (median dose)	One or more of cephalosporins, vancomycin, or rifampin
Berliner [18]	Palacos or Simplex	Palacos (premixed with gentamycin), Simplex (premixed with tobramycin)	Vancomycin (*n* = 61): 7.8 g, Tobramycin (*n* = 65): 8.6 g, Gentamycin (*n* = 29): 2.3 g	Vancomycin (*n* = 57), Cefazolin (*n* = 30), Piperacillin/Tazobactam (*n* = 9), Ceftriaxone (*n* = 5), Daptomycin (*n* = 4), Cefuroxime (*n* = 4), Gentamycin (*n* = 3), Ceftazidime (*n* = 2), Imipenem (*n* = 2), Linezolid (*n* = 2), Oxacillin (*n* = 2), Tobramycin (*n* = 1), Clindamycin (*n* = 1), Nafcillin (*n* = 1), Penicillin (*n* = 1), Cefepime (*n* = 1)
Dagneaux [19]	Plain Simplex	Vancomycin + Gentamycin (*n* = 185), Vancomycin + Tobramycin (*n* = 61), Additional Amphotericin B (*n* = 10)	Vancomycin 3.2 g/40 g, Gentamycin 3.4 g/40 g, Amphotericin B 260 mg/40 g, Tobramycin 3.1 g/40 g	Vancomycin (*n* = 108), Cefazolin (*n* = 51), Ceftriaxone (*n* = 43), Cefepime (*n* = 12), Penicillin (*n* = 11), Ertapenem (*n* = 9), Ciprofloxacin (*n* = 8), Doxycycline (*n* = 8), Daptomycin (*n* = 6), Flagyl (*n* = 4), Cephalexin (*n* = 4), Piperacillin/tazobactam (*n* = 3), Fluconazole (*n* = 3), Gatifloxacin (*n* = 3), Levofloxacin (*n* = 3), Rifampin (*n* = 2), Moxifloxacin (*n* = 2), Gentamicin (*n* = 1), Meropenem (*n* = 1), Trimethoprim-sulfamethoxazole (*n* = 1), Amikacin (*n* = 1), 2 antibiotics (*n* = 32), 3 antibiotics (*n* = 4)
Dagneaux [20]	Plain Simplex	Vancomycin + Gentamycin (*n* = 325), Vancomycin + Tobramycin (*n* = 126), Vancomycin + Daptomycin (*n* = 4), Additional Amphotericin B (*n* = 86)	Vancomycin 3.1 g/40 g, Gentamycin 3.4 g/40 g, Amphotericin B 140 mg/40 g, Tobramycin 3.0 g/40 g, Daptomycin 3.0 g/40 g	Vancomycin (*n* = 184), Cefazolin (*n* = 104), Ceftriaxone (*n* = 61), Cefepime (*n* = 23), Daptomycin (*n* = 23), Ertapenem (*n* = 19), Penicillin (*n* = 13), Levofloxacin (*n* = 13), Fluconazole (*n* = 10), Doxycycline (*n* = 6), Rifampin (*n* = 5), Flagyl (*n* = 3), Piperacillin-tazobactam (*n* = 3), Gentamicin (*n* = 3), Trimethoprim-sulfamethoxazole (*n* = 2), Ciprofloxacin (*n* = 1), Cephalexin (*n* = 1) Moxifloxacin (*n* = 1), Ampicillin/sulbactam (*n* = 1), Antibiotic combination (*n* = 1), 2 antibiotics (*n* = 52), 3 antibiotics (*n* = 8)
Edelstein [21]	Palacos R Bone Cement, Palacos R + G Bone Cement or Cobalt HV Bone Cement	Vancomycin, Tobramycin, Gentamycin	Vancomycin 3.0 g/40 g, Tobramycin 2.4 g/40 g, Gentamycin 0.5 g/41.1 g	6 weeks culture specific treatment
Geller [22]	Low viscosity cement with tobramycin	Vancomycin + Tobramycin (*n* = 199), Vancomycin + Gentamycin (*n* = 32)	Vancomycin 2.65/PMMA bag, Tobramycin 2.6 g/PMMA bag, Gentamycin 1.3 g/PMMA bag (mean doses), Amphotericin B 1 g, Daptomycin 2 g, Cefazolin 10 g, Cefazolin 4 g, Cefazolin 1 g, Ceftazidime 4 g	Vancomycin (*n* = 111), Daptomycin (*n* = 52), Cefazolin (*n* = 45), Zosyn (*n* = 21), Ceftriaxone (*n* = 18), Ciprofloxacin (*n* = 17), Penicillin group antibiotics (*n* = 15), Linezolid (*n* = 8), Gentamicin (*n* = 7), Levofloxacin (*n* = 7), Micafungin (*n* = 4), Tigecycline (*n* = 4), Metronidazole (*n* = 3), Fluconazole (*n* = 2), Rifampin (*n* = 2), Meropenem (*n* = 2), Cefepime (*n* = 2), Furosemide (*n* = 2), Ticarcillin (*n* = 1), Ceftazidime (*n* = 1), Aztreonam (*n* = 1), Moxifloxacin (*n* = 1), Unknown (*n* = 12)
Menge [23]	Palacos (*n* = 23), Cobalt (*n* = 17) or Simplex P (*n* = 44)	Vancomycin (*n* = 69), Tobramycin (*n* = 79), Cefotaxime (*n* = 4), Amikacin (*n* = 1), Amphotericin B (*n* = 1)	Vancomycin 4.0 g, Tobramycin 4.8 g, Cefotaxime 7 g, Amikacin 12.5 g, Amphotericin B 0.06 g (median dose)	Aminoglycosides (*n* = 3), Vancomycin (*n* = 52)
Theil [24]	Copal or Palacos	Gentamycin + Clindamycin (Sensitive bacteria), Vancomycin (Culture negative or Resistant bacteria), Meropenem (Gram negative bacteria), Voriconazole or Amphotericin (Fungal infections)	Gentamycin 2 g/40 g, Clindamycin 1 g/40 g, Vancomycin 2 g/40 g, Meropenem 4 g/40 g, Voriconazole 600 mg/40 g, Amphotericin 200 mg/40 g	Aminopenicillins (*n* = 105), Cephalosporins (*n* = 42), Daptomycin (*n* = 19), Clindamycin (*n* = 56), Antifungal (Caspofungin, voriconazole, fluconazole, micafungin) (*n* = 12), Flucloxacillin (*n* = 67), Vancomycin (*n* = 126), Meropenem (*n* = 18), Linezolid (*n* = 76), Fluoroquinolones (*n* = 30)

*PMMA* polymethyl methacrylate

**Table 3:** Definitions, incidence, and risk factors for development of postoperative acute kidney injury

Study	AKI definition	Incidence of AKI *N* (%)	Predictive univariate risk factors identified	OR (95% CI)	Multivariate risk factors	β or hazard ratio (95% CI)
Aeng [17]	KDIGO Cr increase ≥ 50% from baseline within 7 days of surgery	10 (20)	Premanufactured ACS	8.2 (1.1–60)	NR	NR
			Intraoperative Transfusion	32.5 (2.3–454)		
			Postoperative NSAIDs	23.0 (1.3–398)		
Berliner [18]	KDIGO > 50% Cr increase from baseline (within 30 days preoperatively) to ≥ 1.4 mg/dL	11 (15)	Lower preoperative hemoglobin	1.82 (1.12–2.96)	Lower preoperative hemoglobin*	− 3.0 (− 0.01 to − 0.1)
Dagneaux [19]	KDIGO Stage 1: Cr ≥ 1.5 times baseline or increase of ≥ 0.3 mg/dL within 48 h period Stage 2: Cr ≥ 2 times baseline Stage 3: Cr ≥ 3 times baseline or increase to ≥ 4.0 mg/dL or acute dialysis	81 (19) Without preexisting CKD: *n* = 52 (14) With preexisting CKD: *n* = 29 (45)	Without preexisting CKD		Hypertension	0.17 (0.04–0.19)
			BMI ≥ 30 kg/m^2^	1.9 (1.1–3.5)	Perioperative hypovolemia	0.28 (0.16–0.33)
			Hypertension	3.3 (1.7–6.5)	Acute atrial fibrillation	0.13 (0.05–0.37)
			Inflammatory arthritis	2.7 (1.3–5.4)		
			Non-articulating spacer	4.3 (1.1–18.4)		
			> 3.6 g/batch vancomycin	2.7 (1.4–5.3)		
			> 3.6 g/batch aminoglycosides	2.4 (1.3–4.6)		
			Perioperative hypovolemia	5.1 (2.8–9.4)		
			ICU requirement	9 (1.6–58.9)		
			Acute atrial fibrillation	3.6 (1.4–9.5)		
			Urinary tract infection	8.7 (1.9–39.9)		
			With preexisting CKD			
			Diabetes	4.7 (1.6–13.7)		
			Hydronephrosis	0.4 (0.3–0.5)		
			Postoperative hypovolemia	3.4 (1.3–9.1)		
Dagneaux [20]	KDIGO Stage 1: Cr ≥ 1.5 times baseline or increase of ≥ 0.3 mg/dL within 48 h period Stage 2: Cr ≥ 2 times baseline Stage 3: Cr ≥ 3 times baseline or increase to ≥ 4.0 mg/dL or acute dialysis	23 (10) Without preexisting CKD: *n* = 13 (7) With preexisting CKD: *n* = 10 (28)	Without preexisting CKD		No significant predictors of AKI on multivariate regression	NR
			Diabetes	3.7 (1.1–13.2)		
			Inflammatory rheumatism	4.3 (1.1–17.4)		
			Chronic NSAID use	5.1 (1.6–16)		
			Nephrotoxic agents	7.2 (2.1–24.5)		
			Postoperative hypovolemia	7.4 (2.2–25.3)		
			ICU requirement	24.1 (5.8–100.5)		
			Acute atrial fibrillation	87.5 (8.8–865)		
			With preexisting CKD			
			Prostatic disease/surgery	8.7 (1.7–44.9)		
			Bladder tumor/surgery	9.3 (0.8–101.3)		
			Postoperative hypovolemia	6.7 (1.4–30.6)		
Edelstein [21]	RIFLE Risk: increase in Cr 1.5 times baseline or decrease in GFR of 25% Injury: increase in Cr 2 times baseline or decrease in GFR of 50% Failure: increase in Cr 3 times baseline or decrease in GFR of 75% Loss of kidney function: complete loss of kidney function for > 4 weeks	10 (27)	No significant predictors of AKI on univariate regression	NR	No significant predictors of AKI on multivariate regression	NR
Geller [22]	KDIGO Cr ≥ 1.5 times baseline within one week or increase of ≥ 0.3 mg/dL within 48 h period	65 (26)	Age	NR	BMI	1.1 (1.0–1.1)
			Male gender		Baseline hemoglobin (per 1 g/dL decrease)	0.7 (0.5–1.0)
			BMI		Comorbid condition (including one of diabetes mellitus, CKD, cardiovascular disease, and hypertension)	2.7 (1.3–5.6)
			Baseline hemoglobin (per 1 g/dL decrease)			
			Hemoglobin drop			
			Vancomycin dose			
			Tobramycin dose			
			Comorbid condition (including one of diabetes mellitus, CKD, cardiovascular disease, and hypertension)			
			IV vancomycin			
Menge [23]	KDIGO Increase of ≥ 50% in Cr from preoperative baseline (within 30 days of ACS placement) to a level ≥ 1.4 mg/dL	14 (17)	> 4 g vancomycin	6.0 (1.3–27)	NR	NR
			> 4.8 g tobramycin	5.9 (1.4–24)		
Theil [24]	KDIGO Stage 1: Cr ≥ 1.5–1.9 times baseline or increase of ≥ 0.3 mg/dL Stage 2: Cr ≥ 2–2.9 times baseline Stage 3: Cr ≥ 3 times baseline or acute dialysis requirement	95 (33)	Higher median patient age	NR	Higher age	1.0 (1.0–1.1)
			Higher median Charlson comorbidity index		Higher baseline creatinine	1.9 (1.2–3.1)
			Lower preoperative hemoglobin			
			Higher median number of units of blood transfused			

*AKI* acute kidney injury, *OR* odds ratio, *CI* confidence interval, *β* beta coefficient, *Cr* creatinine, *ACS* antibiotic-laden cement spacer, *NSAID* nonsteroidal anti-inflammatory drug, *NR* not reported, *KDIGO* Kidney Disease Improving Global Outcomes, *CKD* chronic kidney disease, *BMI* body mass index, *ICU* intensive care unit, *RIFLE* Risk, Injury, Failure, Loss of kidney function, and End-stage kidney disease, *IV* intravenous

*Multivariate regression for percent change in creatinine

### Risk factors

Perfusion-related factors such as lower preoperative hemoglobin, higher median number of units of blood transfused or intraoperative transfusion, and postoperative hypovolemia were commonly found to have an association with increased risk for AKI. Other commonly reported risk factors included increasing age, perioperative use of nephrotoxic medications such as nonsteroidal anti-inflammatory drugs, and comorbidity burden (CKD, diabetes mellitus, hypertension, cardiovascular disease, etc.). Six studies performed multivariate analysis along with univariate analysis, while two studies only performed univariate analysis [17, 23]. One study found no predictors associated with AKI risk on either univariate or multivariate analysis [21], while another found several univariate predictors but none after multivariate analysis [19]

### ACS antibiotic concentration

Two studies reported thresholds of antibiotic concentration in ACS associated with increased AKI risk. One found that > 3.6 g of vancomycin or aminoglycosides per batch of cement in first-stage knee revisions was associated with increased AKI risk [20]), while another demonstrated > 4 g of vancomycin or > 4.8 g of tobramycin in an ACS was associated with increased risk [23]. The remaining six studies found no associations with antibiotic dose in ACS and AKI [17–19, 21, 22, 24].

### Quality appraisal

The results of quality appraisal for the included studies are presented in Table 4.

**Table 4:** Quality appraisal of eight studies assessing rates of acute kidney injury in first-stage revision arthroplasty involving the use of antibiotic cement spacers

Study	Q1	Q2	Q3	Q4	Q5	Q6	Q7	Q8	Q9	Q10	Q11	Q12	Total
Aeng [17]	2	2	2	1	0	2	1	0	2	2	2	2	18
Berliner [18]	2	0	2	1	0	2	2	0	NC	NC	NC	NC	9
Dagneaux [19]	2	2	2	2	0	2	2	0	2	2	1	2	19
Dagneaux [20]	2	2	2	2	0	2	2	0	2	2	1	2	19
Edelstein [21]	2	0	2	2	0	2	2	0	2	2	2	2	18
Geller [22]	2	2	2	2	0	2	0	0	2	2	1	2	17
Menge [23]	1	2	2	2	0	2	0	0	2	2	2	2	17
Theil [24]	1	1	2	1	0	2	1	0	NC	NC	NC	NC	8

Methodological items for non-randomized studies for the eight studies included in this systematic review. Comparative studies were assessed using the full 12 question scale; non-comparative (NC) studies were assessed using only the first 8 questions

Q1: A clearly stated aim: the question addressed should be precise and relevant in the light of available literature

Q2: Inclusion of consecutive patients: all patients potentially fit for inclusion (satisfying the criteria for inclusion) have been included in the study during the study period (no exclusion or details about the reasons for exclusion)

Q3: Prospective collection of data: data were collected according to a protocol established before the beginning of the study

Q4: Endpoints appropriate to the aim of the study: unambiguous explanation of the criteria used to evaluate the main outcome which should be in accordance with the question addressed by the study. Also, the endpoints should be assessed on an intention-to-treat basis

Q5: Unbiased assessment of the study endpoint: blind evaluation of objective endpoints and double-blind evaluation of subjective endpoints. Otherwise the reasons for not blinding should be stated

Q6: Follow-up period appropriate to the aim of the study: the follow-up should be sufficiently long to allow the assessment of the main endpoint and possible adverse events

Q7: Loss to follow-up less than 5%: all patients should be included in the follow-up. Otherwise, the proportion lost to follow-up should not exceed the proportion experiencing the major endpoint

Q8: Prospective calculation of the study size: information of the size of detectable difference of interest with a calculation of 95% confidence interval, according to the expected incidence of the outcome event, and information about the level for statistical significance and estimates of power when comparing the outcomes

Q9: An adequate control group: having a gold standard diagnostic test or therapeutic intervention recognized as the optimal intervention according to the available published data

Q10: Contemporary groups: control and studied group should be managed during the same time period (no historical comparison)

Q11: Baseline equivalence of groups: the groups should be similar regarding the criteria other than the studied endpoints. Absence of confounding factors that could bias the interpretation of the results

Q12: Adequate statistical analyses: whether the statistics were in accordance with the type of study with calculation of confidence intervals or relative risk

## Discussion

Acute kidney injury (AKI) has been shown to increase morbidity and mortality following revision TJA. The incidence of AKI after the first stage of a two-stage revision arthroplasty in the current literature ranges between 10 and 33%. Although current evidence is limited and precludes meta-analysis, the most commonly associated risk factors reported include perfusion-related factors (low hemoglobin, perioperative transfusion, hypovolemia), older age, increased comorbidity burden, and perioperative nephrotoxic drug use. Only two studies found that certain antibiotic concentration thresholds in ACS were linked to increased AKI risk, but neither of these were the result of multivariate analysis accounting for other risk factors.

A similar systematic review performed by Luu et al. in 2013 on ten studies consisting of 544 patients reported an AKI incidence of approximately 5% [25], a substantially lower figure than the 21% reported in our study. One possible reason for this discrepancy is the difference in definition of AKI. The current study restricted inclusion criteria to studies using either KDIGO or RIFLE definitions for AKI, whereas the review performed by Luu et al. did not have such restrictions, including many studies that did not specify a threshold. Additionally, of the ten studies in their review all were performed in 2012 or earlier, while the studies included in the current review were performed between 2012 and 2021. Finally, the current review included 1483 total combined revision hip and knee cases, approximately 900 greater than the Luu et al. study. Other recent studies provide similar rates of AKI after ACS placement—a retrospective review conducted in 2018 by Yadav et al. of 3,218 revision TJA found a 29% incidence of AKI after ACS implantation, compared to 3% in aseptic revision cases [26]. AKI in the first stage of revision arthroplasty is an underreported aspect of PJI management in current literature and is a uniquely widespread issue for this patient population, especially when compared to primary or aseptic revision TJA.

In assessing the risk factors for AKI following ACS implantation, the most commonly reported were related to poor perfusion, such as low hemoglobin, and perioperative blood transfusions or hypovolemia. These are well-established risk factors in primary TJA literature, but it is important to recognize the increased rates of blood loss and transfusion with septic revision arthroplasty [27, 28]. This may be due to increased bony work required when explanting prostheses (particularly with the removal of cemented components), edematous and inflamed tissue in the setting of infection, or prolonged operative time. Other risk factors identified in the review of selected studies included use of nephrotoxic agents such as NSAIDs, increasing age, or an increased burden of medical comorbidities such as CKD, diabetes mellitus, or hypertension. These risk factors are prevalent in this population, and it is important for patients to be medically optimized prior to undergoing revision arthroplasty. A review of comorbidities in TKA patients at risk for postoperative mortality and PJI using the Medicare database by Bozic et al. demonstrated that 27% of PJI patients have heart disease, 22% have diabetes, and 70% have hypertension, a major risk factor for developing CKD [29]. Other potential avenues to reduce AKI risk perioperatively could involve the use of single-stage revision in select patients, as this would avoid the implantation of an ACS and involve decreased blood loss [30]. While two-stage revisions are widely referred to as the gold standard of treating PJI, several recent meta-analyses suggest that single-stage revisions may have similar success [8, 31–33]. However, further study is warranted to identify the risk of AKI solely attributed to an ACS. Additionally, while restrictive transfusion protocols are being used widely for primary TJA [34, 35], it may be worth weighing the benefits of a less restrictive policy in a population receiving several nephrotoxic agents to avoid a state of hypoperfusion.

Regarding the ideal concentration of antibiotics in an ACS, this review included two studies that demonstrated ACS antibiotic concentration thresholds associated with increased risk of AKI [19, 20]. Dagneaux et al. found that ACS concentrations > 3.6 g of vancomycin or aminoglycoside were associated with an increased risk of AKI in a retrospective review of 455 cases involving the first stage of revision knee arthroplasty [20]. Furthermore, they found that the nephrotoxic characteristics of these antibiotics worked synergistically as AKI rates were higher when using both when compared to one or the other. In a retrospective review of 84 cases of revision TKA, Menge et al. demonstrated that ACS containing > 4.0 g of Vancomycin or > 4.8 g of Tobramycin had a statistically significant association with the development of an AKI (100% of patients were found to have AKI post-operatively) [23]. Both studies only reported these associations of ACS concentrations with AKI on univariate analysis, however, and the remaining six studies in this review were not able to demonstrate any association with ACS concentration. Additionally, in the Dagneaux et al. paper, the association between spacer antibiotic dosage and AKI rate was not statistically significant after accounting for other factors in a multivariate analysis. In a retrospective review of 91 hip revisions and 156 knee first-stage revisions, Geller et al. demonstrated that prefabricated ACS were associated with increased risk of AKI but did not specify the antibiotic concentration in the spacers [22]. A difficult aspect of identifying AKI risk and the role of ACS concentrations in these cases as there are several perioperative factors such as nephrotoxic agents, preexisting comorbidities, differing degrees of blood loss, and systemic antibiotics that may all play a role. Additionally, the different concentrations of antibiotics used in various studies may also have affected the study outcomes. Even with higher concentrations of spacer antibiotic dosages, several studies like those performed by Aeng et al. and Theil et al. still were unable to demonstrate an association between concentration and AKI risk [17, 24]. Finally, there is a potentially enormous bias in the presence of systemic antibiotic use, as many of these carry substantial nephrotoxic side effects [36, 37]. This is well discussed in the literature, and while high doses of local antibiotics present in spacers may provide a contributing factor there is scarce literature on how this may contribute to kidney injury. The ideal dosage to add to a cement spacer is also controversial, as amount of antibiotic added can also contribute to mechanical weakening of the spacer as well [38]. Further study is warranted to determine safe thresholds, particularly in patients with poor preoperative kidney function. Patients with underlying CKD were found to have nearly a five-fold increase in AKI on CKD compared to those without renal issues preoperatively [23]. Orthopedic surgeons and medical clinicians alike need to be mindful of the type and amount of antibiotic used when trying to safely eradicate infection in these patients.

A significant limitation of the current review was the heterogeneity of the included studies with regards to ACS type, concentrations, concomitant systemic antibiotics, and study design. This variation unfortunately did not allow for meta-analysis. Additionally, AKI is a multifactorial diagnosis—it is difficult to state a cause–effect relationship regarding systemic antibiotic use in two-stage revision. Strengths of this review include the strict inclusion criteria when screening studies, particularly the requirement for a clear and widely recognized definition for AKI. Additionally, revision TJA for PJI is difficult to study in volume, as coding for these procedures is notoriously inaccurate making database studies unreliable [39]. Large databases may not have access to more granular data such as ACS use, antibiotics included in the ACS, or systemic antibiotics. Thus, analysis of these risk factors is limited to level 2 and 3 studies such as those included in this review.

## Conclusion

The high incidence rates of AKI following the first stage of two-stage revision arthroplasty must be considered by providers in the treatment of chronic PJI. Identification of associated risks should be used to better guide preoperative optimization and treatment of these patients, such as reducing the risk of perioperative hypovolemia. Further study is warranted to identify the AKI risk attributed to ACS versus other potential causes of nephrotoxicity as well as the ideal dosage of antibiotics added to ACS constructs.

## Data Availability

The data used for this study were obtained via publicly accessible publications.

## Appendix: Search terms

(((((((arthroplasty[Title/Abstract]) OR (knee replacement[Title/Abstract])) OR (hip replacement[Title/Abstract])) OR (total joint[Title/Abstract])) OR (total knee[Title/Abstract])) OR (total hip[Title/Abstract])) OR (revision arthroplasty[Title/Abstract])) OR (two-stage exchange[Title/Abstract])

AND

((((((((creatinine[Title/Abstract]) OR (acute kidney injury[Title/Abstract])) OR (KDIGO[Title/Abstract])) OR (RIFLE[Title/Abstract])) OR (Kidney Disease Improving Global Outcomes[Title/Abstract])) OR (Risk, Injury, Failure, Loss of kidney function,[Title/Abstract] AND End-stage kidney disease[Title/Abstract])) OR (nephrotoxicity[Title/Abstract])) OR (blood urea nitrogen[Title/Abstract])) OR (aki[Title/Abstract])

AND ((((((((((prosthetic joint infection[Title/Abstract]) OR (PJI[Title/Abstract])) OR (antibiotic spacer[Title/Abstract])) OR (antibiotic-impregnated bone cement spacer[Title/Abstract])) OR (bone cement[Title/Abstract])) OR (periprosthetic joint infection[Title/Abstract])) OR (antibiotic cement spacer[Title/Abstract])) OR (antibiotic-loaded bone cement[Title/Abstract])) OR (tobramycin[Title/Abstract])) OR (vancomycin[Title/Abstract])) OR (cement spacer[Title/Abstract])

882 results in PubMed

Search run 1/21/2022

## References

[1] Schwartz AM, Farley KX, Guild GN, Bradbury TL (2020). Projections and epidemiology of revision hip and knee arthroplasty in the United States to 2030. J Arthroplasty.

[2] Postler A, Lützner C, Beyer F, Tille E, Lützner J (2018). Analysis of total knee arthroplasty revision causes. BMC Musculoskelet Disord.

[3] Barton CB, Wang DL, An Q, Brown TS, Callaghan JJ, Otero JE (2020). Two-stage exchange arthroplasty for periprosthetic joint infection following total hip or knee arthroplasty is associated with high attrition rate and mortality. J Arthroplasty.

[4] Fischbacher A, Borens O (2019). Prosthetic-joint infections: mortality over the last 10 years. J Bone Jt Infect.

[5] Ahmed SS, Haddad FS (2019). Prosthetic joint infection. Bone Joint Res.

[6] Lazic I, Scheele C, Pohlig F, von Eisenhart-Rothe R, Suren C (2021). Treatment options in PJI—Is two-stage still gold standard?. J Orthop.

[7] Charette RS, Melnic CM (2018). Two-stage revision arthroplasty for the treatment of prosthetic joint infection. Curr Rev Musculoskelet Med.

[8] Goud AL, Harlianto NI, Ezzafzafi S, Veltman ES, Bekkers JEJ, van der Wal BCH (2021). Reinfection rates after one- and two-stage revision surgery for hip and knee arthroplasty: a systematic review and meta-analysis. Arch Orthop Trauma Surg.

[9] Sigmund IK, Winkler T, Önder N, Perka C, Renz N, Trampuz A (2019). Complications of resection arthroplasty in two-stage revision for the treatment of periprosthetic hip joint infection. J Clin Med.

[10] Siddiqi A, White PB, Etcheson JI, George NE, Gwam CU, Mistry JB (2017). Acute kidney injury after total knee arthroplasty: a clinical review. Surg Technol Int.

[11] Thongprayoon C, Kaewput W, Thamcharoen N, Bathini T, Watthanasuntorn K, Salim SA (2019). Acute kidney injury in patients undergoing total hip arthroplasty: a systematic review and meta-analysis. J Clin Med..

[12] Levy EM, Viscoli CM, Horwitz RI (1996). The effect of acute renal failure on mortality. A cohort analysis. JAMA.

[13] Moher D, Liberati A, Tetzlaff J, Altman DG, Group* P. Preferred reporting items for systematic reviews and meta-analyses: the PRISMA statement. Ann Intern Med. 2009;151(4):264–9. 10.7326/0003-4819-151-4-200908180-00135 19622511

[14] Khwaja A (2012). KDIGO clinical practice guidelines for acute kidney injury. Nephron Clin Pract.

[15] Ricci Z, Cruz D, Ronco C (2008). The RIFLE criteria and mortality in acute kidney injury: a systematic review. Kidney Int.

[16] Slim K, Nini E, Forestier D, Kwiatkowski F, Panis Y, Chipponi J (2003). Methodological index for non-randomized studies (MINORS): development and validation of a new instrument. ANZ J Surg.

[17] Aeng ES, Shalansky KF, Lau TT, Zalunardo N, Li G, Bowie WR (2015). Acute kidney injury with tobramycin-impregnated bone cement spacers in prosthetic joint infections. Ann Pharmacother.

[18] Berliner ZP, Mo AZ, Porter DA, Grossman JM, Hepinstall MS, Cooper HJ (2018). In-hospital acute kidney injury after TKA revision with placement of an antibiotic cement spacer. J Arthroplasty.

[19] Dagneaux L, Limberg AK, Osmon DR, Leung N, Berry DJ, Abdel MP (2021). Renal toxicity associated with resection and spacer insertion for chronic hip PJI. J Arthroplasty.

[20] Dagneaux L, Limberg AK, Osmon DR, Leung N, Berry DJ, Abdel MP (2021). Acute kidney injury when treating periprosthetic joint infections after total knee arthroplasties with antibiotic-loaded spacers: incidence, risks, and outcomes. J Bone Joint Surg Am.

[21] Edelstein AI, Okroj KT, Rogers T, Della Valle CJ, Sporer SM (2018). Nephrotoxicity after the treatment of periprosthetic joint infection with antibiotic-loaded cement spacers. J Arthroplasty.

[22] Geller JA, Cunn G, Herschmiller T, Murtaugh T, Chen A (2017). Acute kidney injury after first-stage joint revision for infection: risk factors and the impact of antibiotic dosing. J Arthroplasty.

[23] Menge TJ, Koethe JR, Jenkins CA, Wright PW, Shinar AA, Miller GG, et al. Acute kidney injury after placement of an antibiotic-impregnated cement spacer during revision total knee arthroplasty. J Arthroplasty. 2012;27(6):1221-7.e1–2. 10.1016/j.arth.2011.12.005 22321301

[24] Theil C, Riegel RF, Gosheger G, Schwarze J, Schmidt-Braekling T, Moellenbeck B (2021). Acute renal failure after the first stage of a 2-stage exchange for periprosthetic joint infection. J Arthroplasty.

[25] Luu A, Syed F, Raman G, Bhalla A, Muldoon E, Hadley S (2013). Two-stage arthroplasty for prosthetic joint infection: a systematic review of acute kidney injury, systemic toxicity and infection control. J Arthroplasty.

[26] Yadav A, Alijanipour P, Ackerman CT, Karanth S, Hozack WJ, Filippone EJ (2018). Acute kidney injury following failed total hip and knee arthroplasty. J Arthroplasty.

[27] Rao SS, Chaudhry YP, Hasan SA, Puvanesarajah V, Amin RM, Oni JK (2021). Factors associated with perioperative transfusion in lower extremity revision arthroplasty under a restrictive blood management protocol. J Am Acad Orthop Surg.

[28] Boddapati V, Fu MC, Mayman DJ, Su EP, Sculco PK, McLawhorn AS (2018). Revision total knee arthroplasty for periprosthetic joint infection is associated with increased postoperative morbidity and mortality relative to noninfectious revisions. J Arthroplasty.

[29] Bozic KJ, Lau E, Kurtz S, Ong K, Berry DJ (2012). Patient-related risk factors for postoperative mortality and periprosthetic joint infection in medicare patients undergoing TKA. Clin Orthop Relat Res.

[30] Sharqzad AS, Cavalheiro C, Zahar A, Lausmann C, Gehrke T, Kendoff D (2019). Blood loss and allogeneic transfusion for surgical treatment of periprosthetic joint infection: a comparison of one- vs. two-stage exchange total hip arthroplasty. Int Orthop.

[31] Lange J, Troelsen A, Thomsen RW, Søballe K (2012). Chronic infections in hip arthroplasties: comparing risk of reinfection following one-stage and two-stage revision: a systematic review and meta-analysis. Clin Epidemiol.

[32] Kunutsor SK, Whitehouse MR, Lenguerrand E, Blom AW, Beswick AD (2016). Re-infection outcomes following one- and two-stage surgical revision of infected knee prosthesis: a systematic review and meta-analysis. PLoS ONE.

[33] Leonard HA, Liddle AD, Burke O, Murray DW, Pandit H (2014). Single- or two-stage revision for infected total hip arthroplasty? A systematic review of the literature. Clin Orthop Relat Res.

[34] Gupta PB, DeMario VM, Amin RM, Gehrie EA, Goel R, Lee KHK (2018). Patient blood management program improves blood use and clinical outcomes in orthopedic surgery. Anesthesiology.

[35] Newman C, Tran P, McGregor S, Bramley D (2018). Patient blood management strategies in total hip and knee arthroplasty. Curr Orthopaed Pract.

[36] Courtney PM, Melnic CM, Zimmer Z, Anari J, Lee GC (2015). Addition of vancomycin to cefazolin prophylaxis is associated with acute kidney injury after primary joint arthroplasty. Clin Orthop Relat Res.

[37] Jiang EX, Gogineni HC, Mayerson JL, Glassman AH, Magnussen RA, Scharschmidt TJ (2017). Acute kidney disease after total hip and knee arthroplasty: incidence and associated factors. J Arthroplasty.

[38] Samelis PV, Papagrigorakis E, Sameli E, Mavrogenis A, Savvidou O, Koulouvaris P (2022). Current concepts on the application, pharmacokinetics and complications of antibiotic-loaded cement spacers in the treatment of prosthetic joint infections. Cureus.

[39] Roof MA, Lygrisse K, Keitel L, Siddiqi A, Emara A, Piuzzi NS (2021). How Accurate Is ICD-10 coding for revision total knee arthroplasty?. J Arthroplasty.

